# Recent advances in single molecule magnetism of dysprosium-metallofullerenes

**DOI:** 10.1039/c8dt05153d

**Published:** 2019-02-13

**Authors:** Lukas Spree, Alexey A. Popov

**Affiliations:** a IFW Dresden , Helmhotzstraße 20 , 01069 Dresden , Germany . Email: l.spree@ifw-dresden.de ; Email: a.popov@ifw-dresden.de

## Abstract

This article outlines the magnetic properties of single molecule magnets based on Dy-encapsulating endohedral metallofullerenes. The factors that govern these properties, such as the influence of different non-metal species in clusterfullerenes, the cage size, and cage isomerism are discussed, as well as the recent successful isolation of dimetallofullerenes with unprecedented magnetic properties. Finally, recent advances towards the organization of endohedral metallofullerenes in 1D, 2D, and 3D ordered structures with potential for devices are reviewed.

## Abstract 2

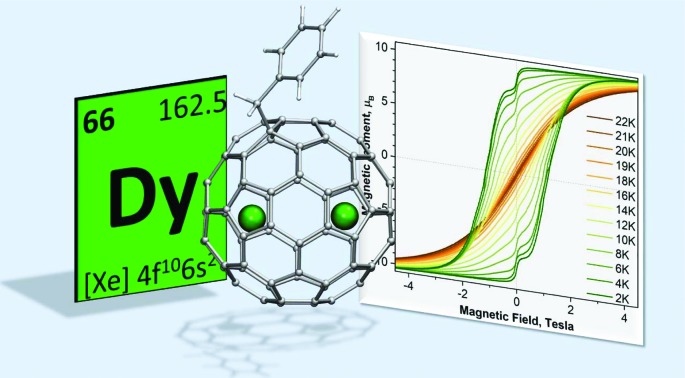
Encapsulation of dysprosium ions in fullerenes results in efficient air stable single molecule magnets, which can be used in preparation of various 1D, 2D, and 3D assemblies.

## Introduction

In 1993 the discovery of quantum tunneling of magnetization in an Mn_12_-complex by Sessoli *et al.* started the ongoing quest to find better single molecule magnets (SMMs).^1^ Better in this case means attaining slower relaxation of magnetization at ever increasing temperatures, since single molecule magnets show great promise toward applications such as high density data storage, quantum computing and spintronics. As the name suggests, the magnetic properties of SMMs are determined from the intramolecular spin structure and are scalable down to the single molecule level. To be able to exhibit SMM properties, a molecule should have a bistable magnetic ground state and a certain energy barrier preventing fast reorientation of the spins in the ground state doublet.†
†It should be noted that all spin systems have some finite relaxation rate, and the threshold between the “fast” relaxation (non-SMM) and “slow” relaxation of magnetization (SMM behaviour) is not well defined. If the molecular material exhibits magnetic hysteresis, it means that its spin relaxation times are on the order of seconds or longer, and the compound is considered to be an SMM (unless hysteresis is caused by intermolecular exchange interactions). Relaxation times of 1–10^–4^ s are usually determined by AC magnetometry, and molecules are still considered as SMMs if the out-of-phase dynamic susceptibility χ′′ is delectable at frequencies lower than 10^3^–10^4^ Hz. Furthermore, the spin relaxation time strongly depends on temperature, and when the latter is sufficiently low, all molecular magnets may exhibit slow relaxation. Practically, conventional magnetometers usually have the lowest temperature limit of 1.8 K. The energy barrier is strongly related to magnetic anisotropy, which therefore is a cornerstone of the SMM field. The first SMMs were multinuclear complexes of transition metals, such as Mn or Fe. Since the beginning of the 2000s, lanthanides have been recognized as viable building blocks of SMMs due to their strong single-ion anisotropy,^2^ and Dy has become the most popular metal for creating new SMMs. The highest temperature of magnetic hysteresis in SMMs exceeding the liquid nitrogen temperature has recently been achieved in Dy-metallocenium salts.^3^

Endohedral metallofullerenes (EMFs) with lanthanide ions entered the field in 2012 when single molecule magnetism was proven for DySc_2_N@C_80_.^4^ Fullerenes facilitate stabilization and protection from ambient conditions of otherwise impossible or unstable molecular configurations within the confines of their carbon cages. The magnetic anisotropy necessary for single molecule magnetism in lanthanide EMFs can be provided by negatively charged nonmetallic species inside the cage and the carbon atoms of the negatively charged fullerene cages themselves. Fullerenes are stable in air under ambient conditions. They feature high thermal stability, allowing evaporation under high-vacuum conditions and growth of thin films *via* sublimation. Besides, EMFs exhibit rich addition chemistry, allowing modification of the cage surface with various functional groups without disrupting the structure of the endohedral species.^5^ This combination of physical and chemical properties, the tunability of the structure of the endohedral magnetic species and a possibility to create functional materials makes EMF-SMMs attractive objects for research. In this frontier, we provide an overview on the recent advances in single molecule magnetism of Dy-containing EMFs. First, we will give a brief overview of the general aspects of synthesis and magnetic characterization of EMFs, and then proceed with the description of different EMF-SMM families, as well as the studies of 1D, 2D, and 3D assemblies of EMF-SMMs.

## Synthesis and structures of EMFs

The preparation of endohedral metallofullerenes usually starts with the so-called Krätschmer–Huffmann synthesis,^6^ modified to suit the requirements of the desired system. This means arc discharge evaporation of graphite electrodes which are filled with a precursor (usually metal or metal oxide) of the desired endohedral species. The evaporation takes place at currents around 100 A at a pressure around 100 mbar under a He atmosphere. Reactive gases (NH_3_, CH_4_, *etc*.) or addition of organic compounds may be employed depending on the specifically desired fullerene system.^7^

As fullerenes are soluble in various organic solvents, in the next step they are extracted from the soot produced from arc discharge evaporation, usually by Soxhlet extraction or boiling under reflux in a suitable solvent. The dissolved fullerenes can be separated by High Performance Liquid Chromatography (HPLC) in multiple steps. Separation is usually the most time-consuming step as the arc discharge evaporation may yield hundreds of different species. Still, isomerically pure compounds are attainable through the use of specialized HPLC columns. Yields are the downside of the otherwise very remarkable fullerene systems, ranging somewhere in milligrams to tens of milligrams of pure compounds produced per year. Molecular structure elucidation of isolated EMFs is then accomplished with conventional approaches such as single-crystal X-ray diffraction, or various spectroscopic techniques.

**Fig. 1:**
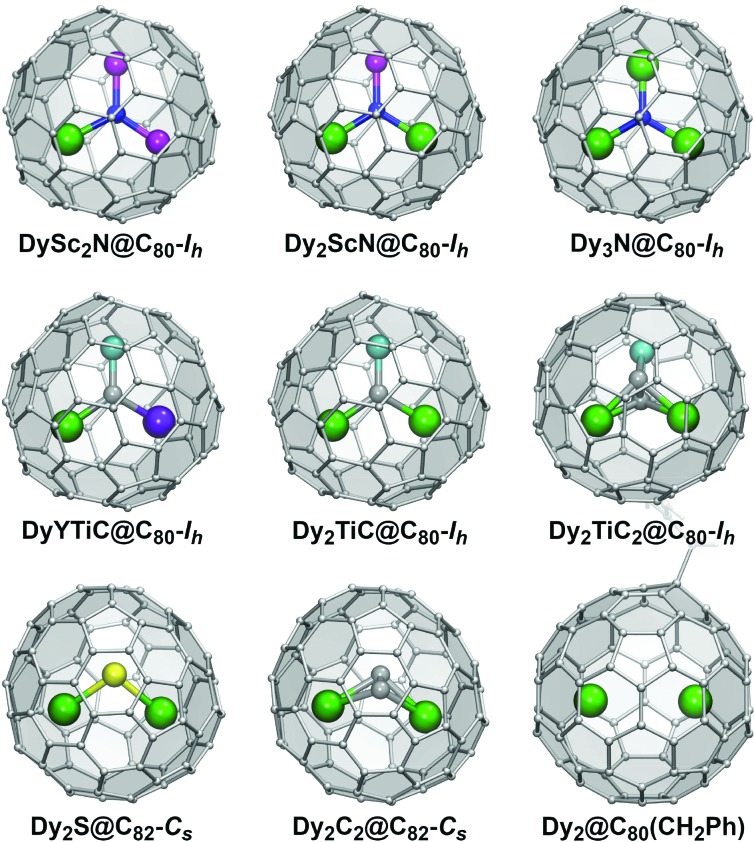
Molecular structures of selected Dy metallofullerenes showing single molecule magnetism. Dy is shown in green, Sc – magenta, Ti – cyan, Y – violet, N – blue, C – gray, and S – yellow. Only a part of the benzyl group of Dy_2_@C_80_(CH_2_Ph) can be seen.

Based on the composition of the endohedral species, EMFs can be classified into two large groups. Conventional EMFs have only metal atoms inside the carbon cage; depending on the number of metal atoms they can be mono-, di-, or trimetallofullerenes. In clusterfullerenes the endohedral species also include non-metal atoms such as C, N, S, or O. The interactions within EMF molecules have a considerable ionic character as metal atoms transfer their valence electrons to the fullerene cage. In clusterfullerenes, non-metal atoms also bear a substantial negative charge (formally, N^3–^, S^2–^, C_2_^2–^, *etc*.). Molecular structures of representative Dy-EMFs discussed in this review are shown in Fig. 1. Further details on the syntheses, structures and properties of EMFs can be found in a number of comprehensive reviews and monographs.^7^,^8^

## Magnetic characterization of EMFs as SMMs

As the SMM behaviour is rooted in the rate of spin relaxation, all parameters employed to characterize SMMs implicitly involve magneto-kinetic characteristics. The latter may be defined and measured in different ways, which results in a certain ambiguity through the literature and complicates the comparison of different SMMs among each other.‡
‡Comparison of SMM performance for different magnetic molecules requires the use of the same parameters determined with more or less identical experimental settings. *E.g.*, comparing closing temperature of hysteresis for one compound to the *T*_B_ value of another one makes little sense. Furthermore, since characteristic SMM parameters are kinetic, they may show strong dependence on the magnetic field or temperature sweep rate. Comparing the closing temperature of hysteresis can be especially misleading, if two compounds are measured with different sweep rates. A few key characteristics may be employed to characterize EMF-SMMs. The blocking temperature of magnetization, magnetic hysteresis and the relaxation time of magnetization are outlined in this section. For more details on characterization of SMMs the reader may refer to specialized books and reviews listed in ref. 1 and 2.

The blocking temperature of magnetization (*T*_B_) is measured *via* two temperature scans. For one scan, magnetization is measured when the sample is cooled down to the lowest possible temperature in a magnetic field of 0.1–0.2 T. For the other scan the sample is cooled in zero field, then the field is turned on and the temperature dependence of magnetization is measured during the temperature increase. These measurements reveal the point where magnetic relaxation becomes fast on the timescale of the measurement: the two curves coincide above *T*_B_ and deviate below *T*_B_. The curve measured for the zero-field cooled sample usually develops a peak with its maximum at *T*_B_. *T*_B_ is slightly dependent on the rate of the temperature sweep; the values reported by our group and discussed hereafter were measured with a temperature sweep rate of 5 K min^–1^ in a field of 0.2 T. The relaxation time at *T*_B_ defined this way is *ca.* 10 s. The blocking temperature should not be confused with another universal parameter, the 100 seconds blocking temperature (*T*_B100_), which, as the name suggests, marks the temperature at which magnetic relaxation takes 100 s.

Hysteresis curves are measured at fixed temperatures by sweeping the magnetic field between negative and positive values. The form of the magnetization curves obtained can give hints towards the magnetic behavior of the sample. A sudden drop of magnetization at zero magnetic field for instance is a sign of tunneling of magnetization (QTM).^9^ The temperature at which the magnetic hysteresis loop closes may also be used to characterize SMMs, but since it strongly depends on the sweep rate, this definition creates a lot of confusion when the values determined by different groups are compared. When the magnetic sweep rate of 2.9 mT s^–1^ is used (as in many of our studies), the closing temperature of hysteresis is close to *T*_B_ as defined above.

Finally, the relaxation times of magnetization *τ*_m_ (or their inverse, relaxation rates) are measured by magnetizing the sample in an external magnetic field at a fixed temperature and then switching the field to zero or another value. Then the evolution of magnetization can be observed over time, and the decay curve is fitted with an exponential function. Very often, single or even double exponential functions cannot describe the measured decay curves, and stretched exponential fitting is commonly used. This method allows the measurement of *τ*_m_ values longer than 10–100 s. Shorter relaxation times may be accessible *via* AC magnetometry. Unfortunately, the latter requires considerably larger sample amounts than DC magnetometry, and only a few EMF samples have been characterized by this technique.

Analysis of the temperature dependence of relaxation times yields a better understanding of the spin relaxation mechanism, which is important for the design of improved SMMs. The relaxation rate is treated as a sum of rates for different processes:*τ*_m_^–1^(*T*) = *τ*_QTM_^–1^ + *A*(*H*)*T*^n_1_^ + *CT*^n_2_^ + *τ*_0_^–1^exp(–*U*^eff^/*T*)

The first term describes the temperature-independent QTM; the second term corresponds to the single-phonon direct process. *A*(*H*) is field-dependent because the phonon frequency corresponds to the Zeeman energy gap of opposite spins; *n*_1_ = 1 but may deviate when a phonon bottleneck occurs. The third term describes the two-phonon Raman mechanism, and *n*_2_ is typically in the range of 5–9. The last term describes the Arrhenius behavior, usually associated with the Orbach mechanism. *U*^eff^ then corresponds to the energy of the excited spin state involved in the relaxation. Careful analysis of the temperature dependence of *τ*_m_ facilitates the identification of the dominant relaxation mechanisms in different temperature ranges. This phenomenological approach to the relaxation of magnetization in SMMs goes back to the studies of spin–phonon relaxation in paramagnetic salts.^10^ Significant limitations of this theory have been recognized in the SMM community during the last few years,^11^ but more refined approaches are yet under development.

## SMM properties of different EMF families

### Nitride clusterfullerenes

**Fig. 2:**
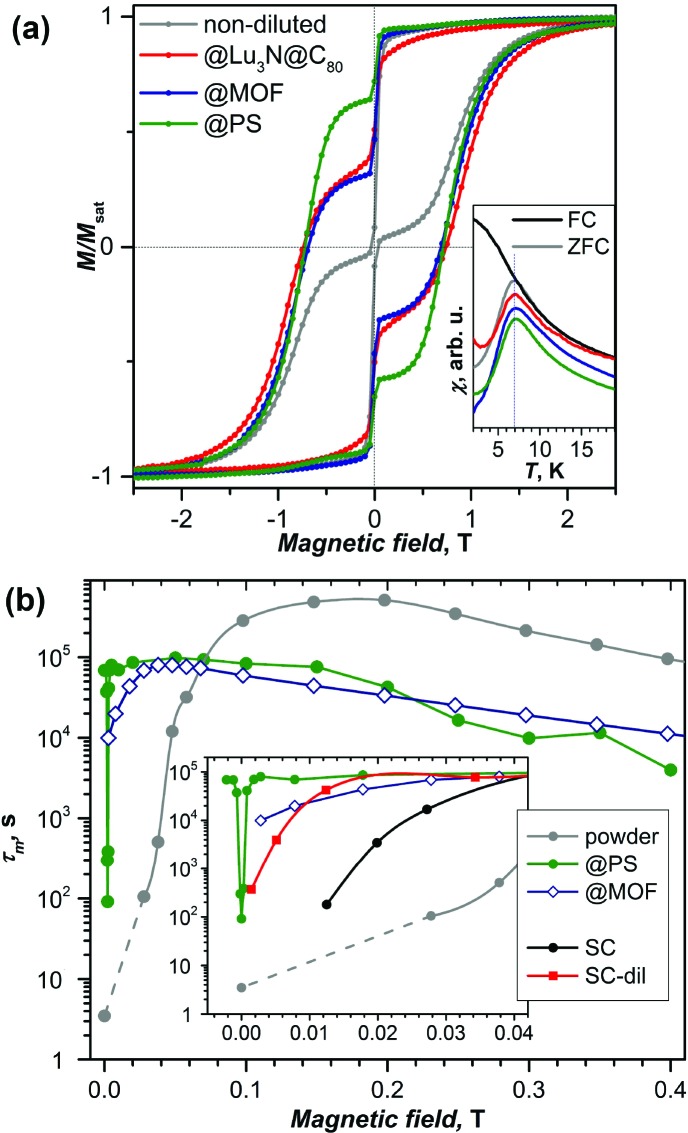
(a) Magnetic hysteresis of non-diluted DySc_2_N@C_80_ powder measured at 1.8 K compared to the sample diluted with the diamagnetic fullerene Lu_3_N@C_80_, absorbed in the metal–organic framework DUT-51(Zr) (@MOF), and dispersed in polymer polystyrene (@PS). Strong variation of the QTM-induced drop of magnetization near zero-field with dilution can be seen. The inset shows that all samples have the same blocking temperature of 7 K. (b) Relaxation times of magnetization measured at 1.8 K in different magnetic fields for non-diluted powder and for diluted samples in MOF, polystyrene (PS) and in a single-crystal (non-diluted, SC, and diluted with Lu_3_N@C_80_, SC-dil). The inset zooms into the small field range. Reproduced from ref. 13.

**Fig. 3:**
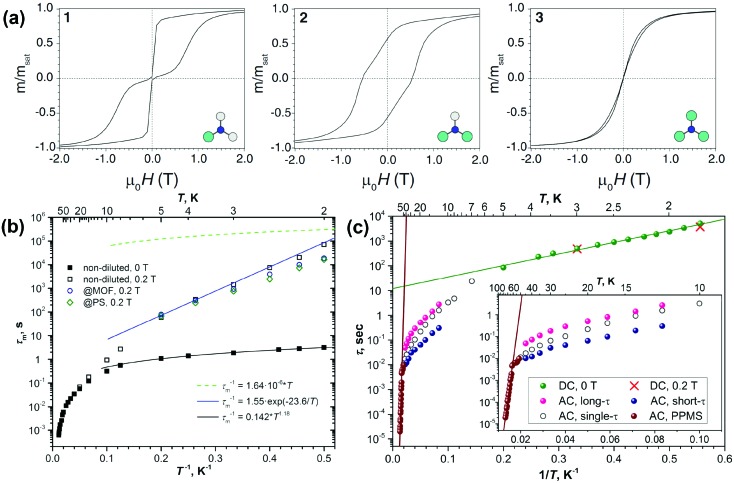
(a) Hysteresis curves for Dy_*x*_Sc_3–*x*_N@C_80_-*I*_h_ (from left to right: *x* = 1, 2, and 3) recorded using SQUID magnetometry at 2 K with a field sweep rate of 0.8 mT s^–1^. Reprinted with permission from Westerström *et al.*, *Phys. Rev. B: Condens. Matter Mater. Phys.*, 2014, **89**, 060406. Copyright 2014 by the American Physical Society. (b) Relaxation times of magnetization of DySc_2_N@C_80_ at temperatures of 2–87 K. Zero-field values are shown as full dots, and in-field (0.2 T) values are denoted as open dots. Relaxation times for non-diluted DySc_2_N@C_80_ are shown in black, and the values for diluted samples are shown in blue (dilution with MOF) and green (diluted with polystyrene, PS). The times longer or shorter than 10 s were determined by DC and AC magnetometry, respectively. The blue line is the fit of the points in the 2–5 K range with the Orbach relaxation mechanism, and the black line represents the fit of the QTM-like zero-field relaxation with the power function of temperature. Reproduced from ref. 13. (c) Relaxation times of the magnetization of Dy_2_ScN@C_80_. Green dots denote the values from DC measurements in zero field; two in-field points (red crosses) are also shown. AC values are measured with MPMS XL (7–50 K; open, magenta, and blue dots) and with PPMS (brown dots, 52–76 K). Magenta and blue dots denote long and short times from double-*τ* fits of the AC data, respectively, and open dots denote single-*τ* fits. Reproduced from ref. 14*b*.

DySc_2_N@C_80_-*I*_h_§
§Since fullerenes have many different isomers, the use of a certain nomenclature is necessary to distinguish different cages. A standard approach is to use Fowler–Manolopoulos spiral algorithm,^43^ which yields a unique number for each cage. In Table 1 we denote fullerene isomers by the formal cage symmetry followed by the spiral number in parenthesis, in the text the spiral numbers are omitted for readability. was the first endohedral fullerene proven to be a single molecule magnet.^4^,^13^ The compound shows hysteresis in SQUID magnetometry measurements up to 6 K and a blocking temperature of *T*_B_ = 7 K (Fig. 2a and 3a). It could be shown that the Dy ion in the compound is responsible for its magnetic properties by comparing the magnetization curves attained by SQUID magnetometry and X-ray magnetic circular dichroism (XMCD) at the Dy M_5_ edge. The peculiar “butterfly shape” of the magnetization curves is attributed to the quantum tunneling of magnetization, the relaxation mechanism common in SMMs with a single magnetic metal ion in the molecule (so-called single-ion magnets). Dilution of the sample with nonmagnetic C_60_ was used to prove that the magnetic properties were indeed a single molecule phenomenon instead of a collective effect. It could also be demonstrated, that dilution increases the relaxation time in zero magnetic field. An in-depth investigation of the relaxation mechanisms in DySc_2_N@C_80_ powders and single-crystals as well as diluted fullerenes in three different diamagnetic matrices was presented in 2018.^13^ Very careful measurements showed a strong influence of dilution on the field dependent relaxation mechanism. For instance, it was shown that strong dilution of the magnetic fullerenes in polystyrene decreases the QTM resonance from 150 mT in an undiluted sample to <1 mT, a feature easily missed in measurements with commercial equipment (Fig. 2). Additionally, measurements of zero field relaxation times revealed a slight temperature dependence of the QTM between 2 and 5 K, which was tentatively attributed to slow energy dissipation through the lattice. Finally, it is noteworthy that the expected linear dependence of log(*τ*_m_) *vs. T*^–1^ (Arrhenius coordinates) could not be confirmed until 87 K, where a low signal to noise ratio of the AC magnetometry data ends the reliable measurement range (Fig. 3).

**Table 1:** *T*_B_ and *T*_B100_ parameters of Dy EMF-SMMs

EMF-SMM	*T*_B_	*T*_B100_	Ref.
DySc_2_N@C_68_-*D*_3_(6140)	3.8	2.3	12
DySc_2_N@C_80_-*I*_h_(7)	7	4.6	4 and 13
DySc_2_N@C_80_-*D*_5h_(6)	5.9	3.6	12

Dy_2_ScN@C_80_-*I*_h_(7)	8	5	14
Dy_2_ScN@C_80_-*D*_5h_(6)	5.3	2.6	12
Dy_2_ScN@C_84_-*C*_s_(51365)	3.3	∼1.8	12

Dy_2_S@C_82_-*C*_3v_(8)	4	2	15
Dy_2_S@C_82_-*C*_s_(6)	∼2		15
Dy_2_S@C_72_-*C*_s_(10528)	<2		15

DyYTiC@C_80_-*I*_h_(7)	7	∼5	16

Dy_2_TiC@C_80_-*I*_h_(7)	∼2	1.7	17
Dy_2_TiC@C_80_-*D*_5h_(6)	∼2		17

Dy_2_TiC_2_@C_80_-*I*_h_(7)	<1.8		17
Dy_2_C_2_@C_82_-*C*_s_(6)	∼2		15

Dy_2_@C_80_(CH_2_Ph)	21.9	18	18

*Ab initio* calculations showed that the Dy ion in the nitride cluster experiences a strong uniaxial ligand field with an overall splitting in the order of 1500 cm^–1^.^19^ The quantization axis is aligned parallel to the Dy–N bond, and the ground state is a Kramers doublet with *J*_*z*_ = ±15/2 separated from higher energy states by *ca.* 400 cm^–1^. Thus, equilibrium magnetic properties of Dy-nitride clusterfullerenes up to room temperature are essentially determined by the ground state doublet. Strong uniaxial anisotropy of lanthanide ions in the LnSc_2_N@C_80_-*I*_h_ molecules was also confirmed by paramagnetic NMR spectroscopy and point-charge ligand-field calculations.^20^ Dy was found to impose the strongest paramagnetic shift on the ^45^Sc nuclear spin in the whole lanthanide series.

The first investigation of the magnetic properties of dinuclear Dy_2_ScN@C_80_-*I*_h_ and trinuclear Dy_3_N@C_80_-*I*_h_ was published in 2014,14*a* followed by a detailed study of temperature dependence in Dy_2_ScN@C_80_ in 2017.14*b* Dy_2_ScN@C_80_ exhibits magnetic hysteresis and blocking of magnetization at 8 K and does not show the QTM present in its single-ion counterpart, DySc_2_N@C_80_. This can be attributed to the ferromagnetic coupling of the two Dy spins in Dy_2_ScN@C_80_. Flipping one of the Dy spins brings the system into an antiferromagnetically coupled state, which is higher in energy than the ground state with ferromagnetic coupling by 10 K. 4.6 K, roughly half of this energy, is attributed to dipolar interactions, and the rest to exchange coupling. This barrier prevents zero-field QTM in Dy_2_ScN@C_80_. Indeed, relaxation times show Arrhenius behavior at low temperature with the *U*^eff^ corresponding to the energy difference between the ferromagnetic and antiferromagnetic states (Fig. 3c), proving that relaxation proceeds *via* the latter state. AC magnetometry was used to investigate magnetic relaxation at higher temperatures and revealed an Orbach mechanism with an exceptionally high thermal barrier of 1735 ± 21 K (Fig. 3c). *Ab initio* calculations helped to assign this barrier to the relaxation *via* the 5^th^ Kramers doublet.

The third member of the series, Dy_3_N@C_80_-*I*_h_, does not show remanence (Fig. 3a), which can be attributed to a frustrated magnetic ground state.14a,19a Ferromagnetic coupling in a triangular Dy_3_N cluster cannot be realized for all three Dy spins at once since the single-ion quantization axes are linked to corresponding Dy–N bonds arranged at 120° with respect to each other.

The promising properties of the Dy_*x*_Sc_3–*x*_N@C_80_-*I*_h_ family led to increased interest in endohedral fullerene SMMs and subsequently many systems were synthesized and checked for their magnetic properties. Basically, three parameters which potentially affected the magnetic properties were identified: the magnetic species themselves, the size or the specific isomer of the encapsulating cage, and the nonmetallic species that might also be encapsulated in the fullerene.

The influence of the carbon cage size and its isomerism on the magnetic properties of encapsulated DySc_2_N and Dy_2_ScN clusters has been studied recently.^12^ DySc_2_N@C_68_-*D*_3_, DySc_2_N@C_80_-*D*_5h_, DySc_2_N@C_80_-*I*_h_, Dy_2_ScN@C_80_-*D*_5h_, Dy_2_ScN@C_80_-*I*_h_ and Dy_2_ScN@C_84_-*C*_s_ were compared for their key characteristic markers. It could be demonstrated that the C_80_-*I*_h_ cage isomer yields the SMM with the highest blocking temperature and slowest relaxation of magnetization. It was hypothesized that free movement of the clusters inside the cage and subsequent week spin–phonon coupling seem to be the strongest factor in enhancing the magnetic properties of EMFs.

### Sulfide clusterfullerenes

**Fig. 4:**
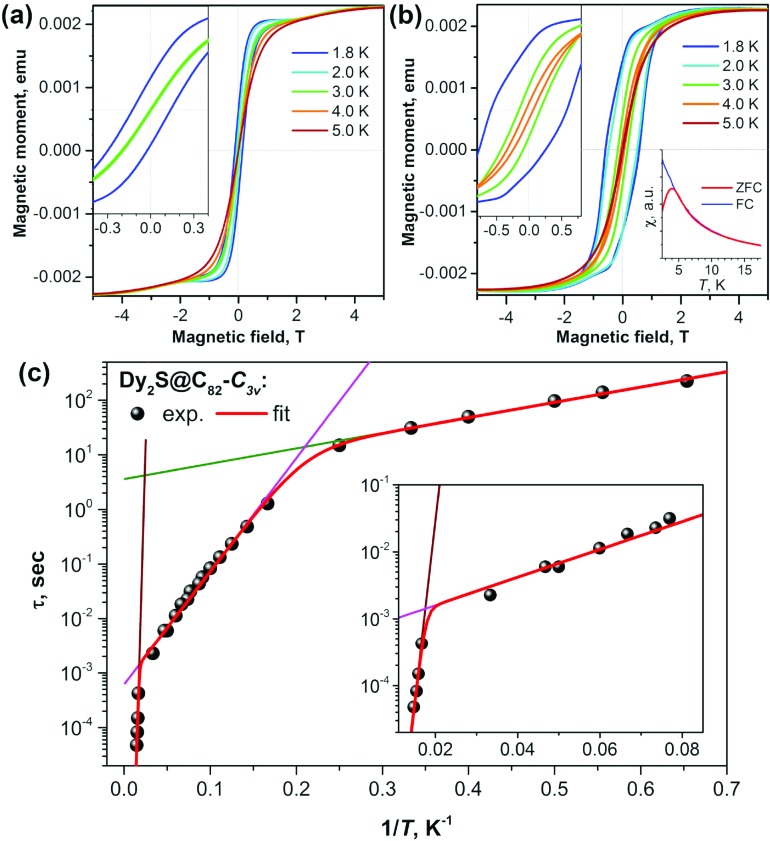
Magnetization curves for (a) Dy_2_S@C_82_-*C*_s_ and (b) Dy_2_S@C_82_-*C*_3v_ measured at *T* = 1.8–5 K with a magnetic field sweep rate of 8.33 mT s^–1^. The inset in each panel zooms into the region near zero-field. The inset in (b) shows the determination of *T*_B_ of Dy_2_S@C_82_-C_3v_ from the peak in the susceptibility of the zero-field cooled sample (magnetic field: 0.2 T, temperature sweep rate: 5 K min^–1^). (c) Magnetization relaxation times of Dy_2_S@C_82_-*C*_3v_; dots are experimental points, red lines are results of a global fit with three Orbach processes; and green, magenta, and brown lines represent contributions of individual Orbach processes. The inset shows an enhancement of the high-temperature range. Reproduced from ref. 15.

As seen in Dy_2_ScN@C_80_, coupling the two Dy atoms *via* a nitride ion leads to the suppression of quantum tunneling of magnetization. Checking different non-metal units in Dy-clusterfullerenes was therefore a logical next step. The sulfide clusterfullerenes Dy_2_S@C_82_-*C*_3v_, Dy_2_S@C_82_-*C*_s_, and Dy_2_S@C_72_-*C*_s_ were synthesized and purified to study this effect.^15^ The sulfide clusterfullerene with *C*_3v_ cage symmetry was found to be the best SMM among the three, showing hysteresis, which closes between 4 and 5 K, and a blocking temperature *T*_B_ of 4 K (Fig. 4). The study revealed considerable cage dependence of the magnetization dynamics. Additionally, in the *C*_3v_ isomer, three Orbach processes with different thermal barriers could be identified (Fig. 4c), governing the relaxation behavior at different temperatures, respectively.

Among the few SMMs with sulfur-ligated Dy reported so far,^21^ Dy_2_S-clusterfullerenes have the longest relaxation times and the highest relaxation barriers. The reason is that in Dy_2_S@C_2*n*_ molecules, sulfur bears a substantially larger negative charge (the formal charge state is S^2–^) and Dy–S distances are at the same time much shorter, which altogether leads to a strong axial ligand field.

### Carbide clusterfullerenes

Clusters featuring a Ti=C double bond and two more metal atoms enable the synthesis of SMMs with up to three different metal species in one cage.^16^,^17^,^22^ This is rare as chromatographic separation becomes more complex with every endohedral species added. Remarkably, it was found that DyYTiC@C_80_-*I*_h_ shows a relatively high blocking temperature of magnetization of 7 K, which is comparable to that of DySc_2_N@C_80_-*I*_h_.^16^ The hysteresis of Dy_2_TiC@C_80_-*I*_h_ on the other hand closes at 3 K, which compares very poorly to that of Dy_2_ScN@C_80_-*I*_h_.^17^ A comparison of dinuclear carbide and nitride clusterfullerenes shows that the anisotropy introduced by the nonmetallic unit plays a smaller role in magnetic behavior at low temperature than the exchange interaction. This becomes even more pronounced in the congener of Dy_2_TiC, Dy_2_TiC_2_, with one more carbon atom in the endohedral cluster, which only shows very narrow hysteresis at 1.8 K.^17^

Another carbide clusterfullerene, Dy_2_C_2_@C_82_-*C*_s_, which is isostructural to the aforementioned sulfide clusterfullerene Dy_2_S@C_82_-*C*_s_, shows similar magnetic properties to the latter.^15^ Fitting of AC magnetometry data revealed barriers of 15.2 K and 17.4 K, respectively, between their ferromagnetic ground states and what is presumed to be an antiferromagnetic excited state. The main difference appears in their respective attempt times *τ*_01_, which are 2.9 ms for Dy_2_S@C_82_-*C*_s_ and 0.5 ms for Dy_2_C_2_@C_82_-*C*_s_, yielding shorter relaxation times for the latter, therefore making it the weaker SMM.

In conclusion, the studies on clusterfullerenes yielded the following insights:

(1) Nitride clusterfullerenes give the best SMMs, followed by sulfide, C_1_-carbide and C_2_-carbide clusterfullerenes in that order.

(2) The non-metal clusters facilitate the strong single-ion anisotropy needed to make SMMs. In dinuclear EMFs they also contribute to the coupling of the magnetic ions, suppressing QTM and thus giving SMMs with pronounced remanence.

(3) The fullerene cage is not just an inert container, but also plays a role in the relaxation of magnetization, as evidenced by the variation in SMM properties for different cage sizes and isomers.

*Ab initio* calculations predicted that oxide clusterfullerenes have the largest crystal field splitting among clusterfullerenes, making them an interesting subject for future investigation.^15^,^23^

Still, the interaction between magnetic ions coupled by nonmetallic atoms is relatively weak, giving an energy barrier between ferromagnetic and antiferromagnetic states of less than 15 K.

### Dimetallofullerenes

To enhance the coupling between magnetic ions a covalent metal–metal bond presents the most elegant solution. Dimetallofullerenes (di-EMFs) proved uniquely suited to this end. In EMFs, lanthanide ions tend to give their valence electrons away and fullerene cages tend to act as electron acceptors. The metal–metal bonding molecular orbital is one of the frontier orbitals in di-EMFs, and its population depends on its energy in relation to the cage MOs.^24^ M_2_@C_82_ di-EMFs (M = Sc, Y, Er, Lu) were found to have occupied M–M bonding MOs with formal charges of +2 on both metal ions. Electrochemical manipulation of the Er–Er bonding orbital in Er_2_@C_82_ was shown to effectively change coupling by creating a three spin system {Er^3+^–*e*–Er^3+^}.^25^

In C_80_-*I*_h_ cages the valence MOs of the La_2_ dimer have a relatively high energy, which leads to all valence electrons being transferred to the fullerene cage and yielding La ions with a formal charge of +3. However, a single-electron reduction of La_2_@C_80_ gives the monoanion with a single-electron La–La bond as evidenced by EPR spectroscopy.^26^ On the other hand, Y_2_ and intermediate lanthanide dimers such as Gd_2_, Dy_2_, or Lu_2_ give only five electrons to the cage, leaving each ion with a formal charge of +2.5 and the highly sought-after single electron bond between them. The downside of this is the formation of radicals, which are hard to extract from the soot due to polymerization in neutral solvents.^27^ Stabilization of this unique electronic configuration can be achieved by the substitution of a carbon atom in the cage by nitrogen, giving azafullerenes M_2_@C_79_N,^28^ or by extraction in a polar solvent such as dimethylformamide (DMF) and subsequent functionalization of the cage with a radical group to form monoadducts M_2_@C_80_-R (R = CF_3_,^27^,^29^ benzyl^18^).

In 2015, a computational study by Sing *et al.* predicted a strong magnetic exchange in Gd_2_@C_79_N and a large magnetization relaxation barrier in Dy_2_@C_79_N.^30^ Indeed, the coupling constant between Gd and the unpaired electron residing on the Gd–Gd bond in Gd_2_@C_79_N has recently been found to be 170–175 cm^–1^.^31^

**Fig. 5:**
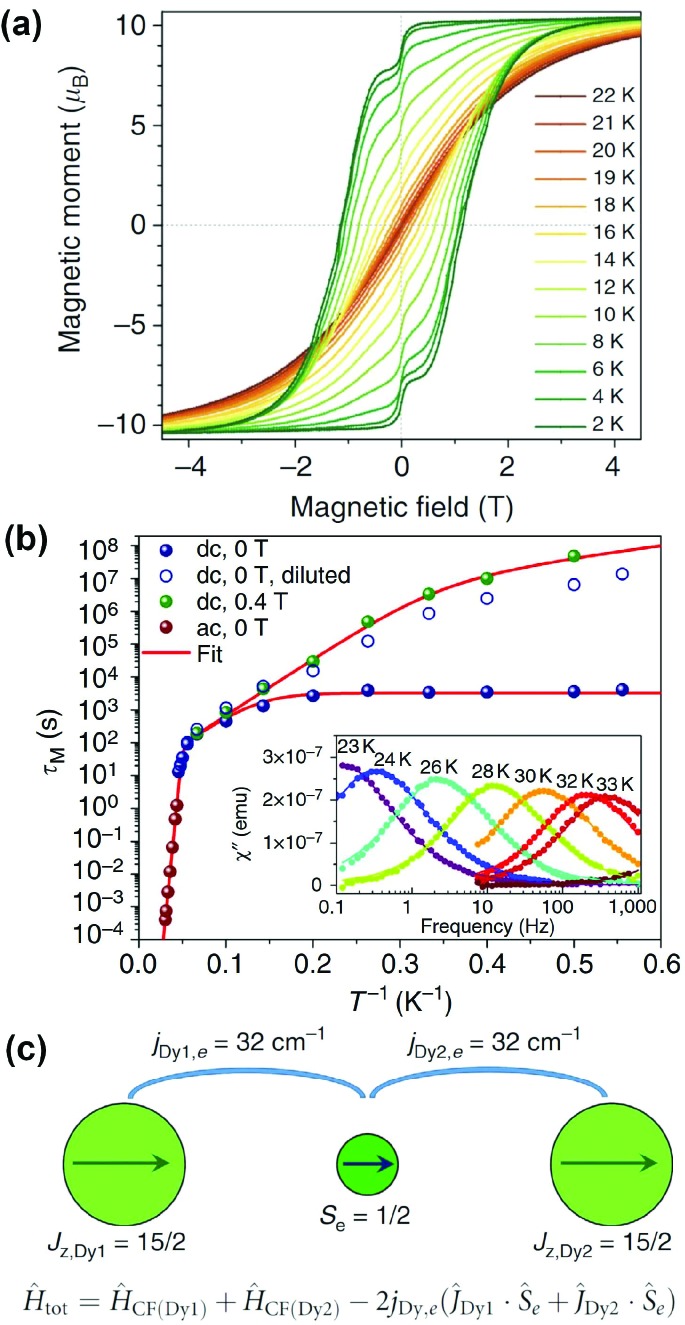
(a) Magnetic hysteresis in Dy_2_@C_80_(CH_2_Ph) between 2 and 22 K, field sweep rate: 2.9 mT s^–1^. (b) Magnetization relaxation times of Dy_2_@C_80_(CH_2_Ph) in zero-field and in a field of 0.4 T. The inset shows the out-of-phase dynamic susceptibility χ′′ measured at different temperatures between 23 and 33 K. (c) Alignment of magnetic moments in the ground state of Dy_2_@C_80_(CH_2_Ph) and respective spin Hamiltonian (CF denotes the crystal field). Reproduced from ref. 18.

Ultimately, the EMF-SMM record set by Dy_2_ScN@C_80_ was broken by a benzyl monoadduct of Dy_2_@C_80_-*I*_h_, Dy_2_@C_80_(CH_2_Ph). Dy_2_@C_80_ could be extracted from soot with DMF, presumably in the form of a monoanion, whereas non-polar solvents such as toluene or carbon disulfide did not work in this regard. Functionalization of the cage with a benzyl group by treatment with benzyl bromide afforded toluene-soluble air-stable molecules which could be isolated by HPLC. An in-depth investigation into its synthesis and properties was published in 2017.^18^ The SMM properties displayed by this molecule are truly remarkable with a blocking temperature of magnetization of 21.9 K and hysteresis observable between 1.8 and 21 K (Fig. 5a). The 100 seconds blocking temperature *T*_B100_ was determined to be 18 K. Relaxation of magnetization in Dy_2_@C_80_(CH_2_Ph) in zero field between 1.8 and 5 K proceeds *via* QTM with a relaxation time of 3257 s. When QTM is quenched by the application of a finite magnetic field, an Orbach-like process, attributed to phonon assisted relaxation, with an effective barrier of 40 K starts at 3 K and becomes dominant between 10 and 18 K. Above 20 K another Orbach process with *U*^eff^ = 613 K takes over (Fig. 5b).

Dy_2_@C_80_(CH_2_Ph) can be treated as a three spin system {Dy^3+^–*e*–Dy^3+^}, with the Dy ions coupling ferromagnetically to the electron from both sides (Fig. 5c). *Ab initio* calculations were used to show that the negative charge between the positively charged ions enforces easy axis anisotropy along the Dy–Dy bond. The direct antiferromagnetic coupling between the Dy ions is very weak and can be neglected. Determination of the coupling constants between the Dy ions and the electron spin between them was achieved by simulating magnetization and *χT* curves to match experimental data, with a very large value of *j*_Dy,e_ = 32 cm^–1^ or 46 K giving the best fit. Assuming this coupling constant, the energy of the exchange excited state, in which one Dy spin is flipped, was calculated to be 613 K. This energy matches the high-temperature Orbach barrier, determined from fitting magnetic relaxation data. The successful isolation of this elusive class of EMFs marks a breakthrough for the field.

## Beyond powder samples

The magnetic properties described in the previous section were obtained for bulk powder EMF samples. This is a first step in magnetic characterization of SMMs, but for the evaluation of their potential applications the studies of powder samples are insufficient. Playing to the strengths of SMMs, addressability of single molecules would be needed, which should be most easily achievable in 1D or 2D arrays. The well-defined positioning in 3D matrices is desirable as well to fine-tune the properties of future nanomaterials.

### 1D arrays: peapods

Single walled carbon nanotubes (SWCNTs) can provide a channel, in which endohedral fullerenes may line up in a 1D chain.^32^ These structures are commonly known as peapods and show great promise for applications in spintronic devices and quantum computation.

**Fig. 6:**
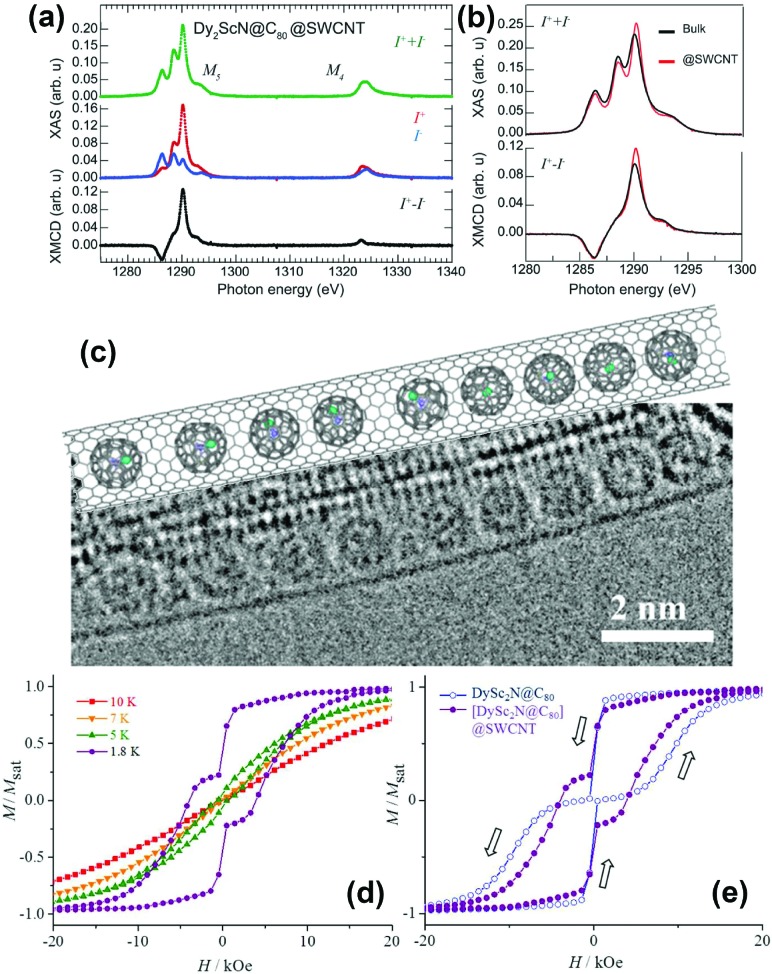
(a) X-ray absorption spectra of Dy_2_ScN@C_80_ encapsulated in SWCNTs recorded using right (*I*^+^) and left (*I*^–^) circularly polarized X-rays. (b) A comparison of the normalized total absorption and XMCD spectra from bulk Dy_2_ScN@C_80_ and Dy_2_ScN@C_80_ encapsulated in SWCNTs. The temperature is 2 K, and an external magnetic field of 6.5 T is applied parallel to the X-ray beam and the surface normal to the samples. (c) TEM image and structural model of the [DySc_2_N@C_80_]@SWCNT peapod. (d) Magnetization curves of [DySc_2_N@C_80_]@SWCNT measured at different temperatures by SQUID magnetometry; (e) comparison of magnetic hysteresis curves for bulk DySc_2_N@C_80_ and [DySc_2_N@C_80_]@SWCNT peapod (*T* = 1.8 K). (a) and (b) reproduced from ref. 33. Reprinted with permission from Nakanishi *et al.*, *J. Am. Chem. Soc.*, 2018, **140**, 10955. Copyright 2018 by the American Chemical Society.

A study on chains of Dy_2_ScN@C_80_ inside SWCNTs by XMCD was published recently by Avdoshenko *et al.*^33^ A comparison of the encapsulated EMFs with a powder sample of the same composition revealed a reduction of the magnetic bistability caused by the encapsulation. Additionally, partial ordering of the clusters was observed (Fig. 6a and b). An explanation of this behavior was given with the help of calculations on isostructural Y_2_ScN@C_80_ packed in SWCNTs. Depending on the relationship between the cage size and diameter of the used SWCNT, energetically preferable orientations of the clusters emerged.

DySc_2_N@C_80_ was encapsulated in SWCNTs by Nakanishi *et al.*^34^ (Fig. 6c). Here hysteresis was conserved and an increase in coercivity and a longer relaxation time compared to those of the powder sample could be observed (Fig. 6d and e). Thus, encapsulation within the SWCNT partially suppressed the QTM relaxation of DySc_2_N@C_80_, and the authors attributed this to a dilution effect which is also observable in bulk powder samples.

### 2D arrays: (sub)monolayers on substrates

Deposition of SMM molecules on conducting surfaces is an obvious route to their addressable 2D arrays.^35^ However, magnetic bistability in monolayers was observed for only a few of the many substances that show SMM behavior in bulk samples. The main difficulties in this route are caused by insufficient thermal or chemical stability of SMM molecules precluding the formation of monolayers and the detrimental effect of molecule–metal interactions on the SMM properties.

**Fig. 7:**
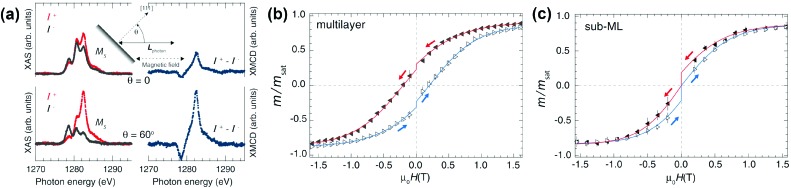
(a) Sub-monolayer (ML) of Dy_2_ScN@C_80_/Rh(111), *T* = 4 K, *μ*_0_*H* = 6.5 T; measurement geometry is shown in the inset. The polarization dependent X-ray absorption spectra (left panel), and the corresponding XMCD spectra (right panel) measured at incidence angles of *θ* = 0° and *θ* = 60°. Strong angular dependence points to the preferential alignment of Dy spins parallel to the surface. (b, c) Hysteresis curves measured by XMCD from a multilayer (b) and a sub-ML (c) of Dy_2_ScN@C_80_/Rh(111) at a magnetic field sweep rate of 2 T min^–1^ and a sample temperature of ∼4 K. The drop in magnetization at zero field is a consequence of the time of 30 s it takes the magnet to switch polarity. Reprinted with permission from Westerström *et al.*, *Phys. Rev. Lett.*, 2015, **114**, 087201. Copyright 2015 by the American Physical Society.

In 2014, Westerström *et al.* published a study of Dy_2_ScN@C_80_ deposited onto a Rh(111) surface by evaporation under vacuum.36*a* In a submonolayer, ordering of the magnetic moments on the surface and hysteresis of magnetization could be observed at 4 K (Fig. 7). The relaxation time was estimated to be approximately 16 times faster than that for powder samples, although demagnetization by X-ray irradiation should be taken into account,^37^ as the results were obtained by XMCD with synchrotron radiation. A recent XMCD study showed that deposition of Dy_2_ScN@C_80_ onto *h*-BN/Rh(111) nanomesh resulted in a broader hysteresis than on a pure Rh(111) surface.36*b*

**Fig. 8:**
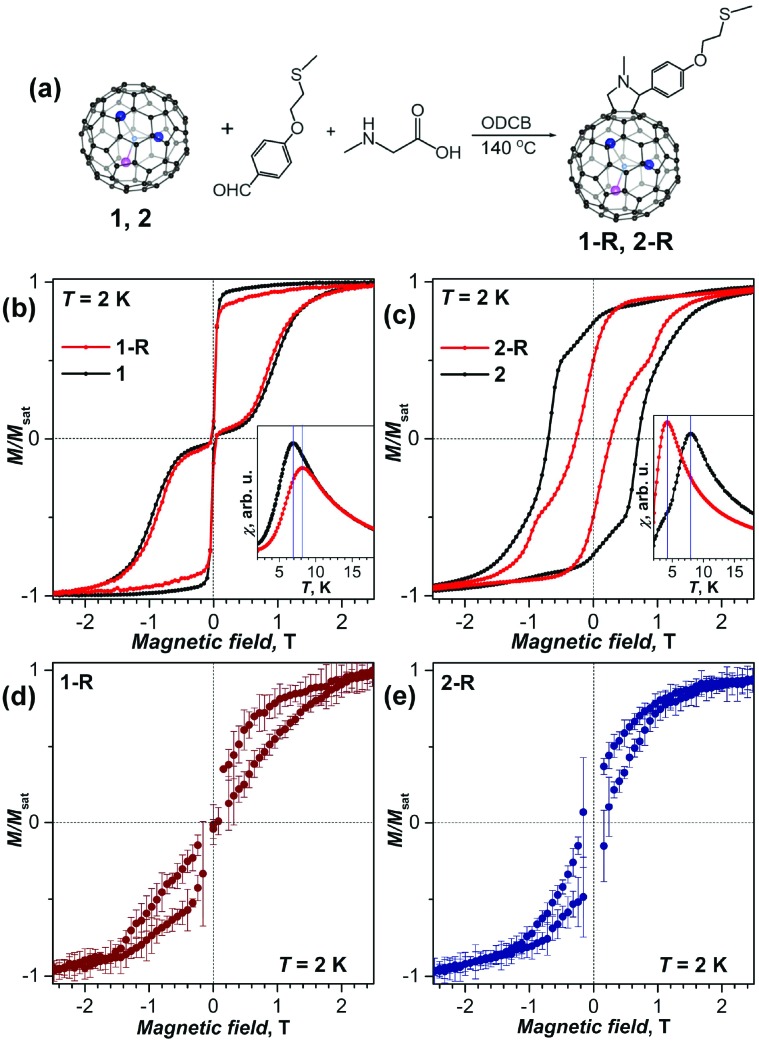
(a) Scheme of a Prato reaction to obtain **EMF-R** derivatives (EMF = DySc_2_N@C_80_ (**1**), and Dy_2_ScN@C_80_ (**2**), **R** denotes the functional group with a thioether linker). (b, c) Magnetization curves of (b) **1-R** and **1**, and (c) **2-R** and **2** measured by SQUID magnetometry at *T* = 2 K (field sweep rate: 2.9 mT s^–1^); the insets show determination of the blocking temperatures of magnetization *T*_B_ (temperature sweep rate: 5 K min^–1^). (d, e) magnetization curves of sub-monolayers of **1-R** (d) and **2-R** (e) on Au(111) measured by XMCD at 2 K with a sweep rate of 2 T min^–1^ (averaging over five measured curves, and error bars are standard deviations). Reproduced from ref. 38.

Chemical functionalization of Dy_2_ScN@C_80_ and DySc_2_N@C_80_ with a thioether group was achieved *via* 1,3-dipolar cycloaddition by Chen *et al.*^38^ The SMM behavior was observed in functionalized EMFs, but the magnetic properties changed noticeably in comparison with those of pristine EMFs (Fig. 8a–c). The blocking temperature *T*_B_ was increased by 1 K for DySc_2_N@C_80_, but decreased by 4 K for Dy_2_ScN@C_80_. The coercive field for functionalized Dy_2_ScN@C_80_ was also visibly lower compared to that of the non-functionalized sample. The functionalized molecules were then able to attach to an Au(111) surface by physisorption. When deposited on gold both functionalized molecules showed hysteresis of magnetization at 2 K as proven by XMCD (Fig. 8d and e). DFT calculations showed that a horizontal configuration of the functionalized molecules, with the fullerenes touching the metal, is energetically favorable over a vertical configuration. Along with X-ray induced demagnetization this might be responsible for the clearly shortened relaxation times. Additionally, the structures are highly mobile at room temperature, leading to random orientations on the surface. Further analysis revealed a certain protective property of the cage π-system for the magnetic state of the cluster, even when the cage interacts strongly with the metal surface.

### 3D arrays: MOFs

Metal organic frameworks (MOFs) provide highly ordered porous structures, which can accommodate SMM molecules.^39^ Wang *et al.* introduced DySc_2_N@C_80_ into the pores of MOF-177 and observed a suppression of QTM.^40^ Similar suppression of zero-field QTM was observed when DySc_2_N@C_80_ was incorporated into the pores of DUT-51(Zr)^13^,^41^ as mentioned above in the discussion of the dilution effect on the QTM of DySc_2_N@C_80_. The distance between EMF molecules in the MOF is considerably longer than those in the powder EMF samples, which leads to much weaker dipolar magnetic fields and hence to a narrowing of the QTM resonance (*i.e.* to the decrease of the field range in which QTM can take place).

Suppression of QTM in DySc_2_N@C_80_ was also observed when it was encapsulated within the pores of an azobenzene-functionalized MOF.^42^ The authors claimed that irradiation of the ^Azo^MOF with light causing *trans*–*cis* isomerization of azobenzene moieties improved the SMM properties of absorbed DySc_2_N@C_80_.

## Concluding remarks

Over the last few years Dy containing EMFs have been proven to comprise robust SMMs. Numerous recent studies have contributed to the understanding of the forces that govern their magnetic properties. Through this understanding, control over anisotropy and intramolecular interactions is attainable, by the choice of non-metal species as well as cage sizes and isomers. While among clusterfullerenes the nitrides show the strongest magnetic properties, followed by sulfides and carbides, oxides may be promising as well. The successful isolation of Dy_2_@C_80_-CH_2_Ph with a single electron bond gives a new direction to the field, providing molecules with the highest blocking temperatures measured for EMF SMMs and among the highest for SMMs in general. The high stability and protection provided by the fullerene cages make for perfect prospects towards use in future devices, as this enhances processability. Initial studies have already shown various routes toward 1D, 2D and 3D structures.

## Conflicts of interest

There are no conflicts to declare.
